# Antimicrobial Activity of Thin Solid Films of Silver Doped Hydroxyapatite Prepared by Sol-Gel Method

**DOI:** 10.1155/2014/165351

**Published:** 2014-01-12

**Authors:** Simona Liliana Iconaru, Patrick Chapon, Philippe Le Coustumer, Daniela Predoi

**Affiliations:** ^1^National Institute of Materials Physics, P.O. Box MG 07, Magurele, Romania; ^2^Horiba Jobin Yvon S.A., 16-18 rue du Canal, 91165 Longjumeau Cedex, France; ^3^Universite Bordeaux, EA 4592 Géoressources & Environnement, ENSEGID, 1 allée F. Daguin, 33607 Pessac Cedex, France

## Abstract

In this work, the preparation and characterization of silver doped hydroxyapatite thin films were reported and their antimicrobial activity was characterized. Silver doped hydroxyapatite (Ag:HAp) thin films coatings substrate was prepared on commercially pure Si disks by sol-gel method. The silver doped hydroxyapatite thin films were characterized by various techniques such as Scanning electron microscopy (SEM) with energy Dispersive X-ray attachment (X-EDS), Fourier transform infrared spectroscopy (FT-IR), and glow discharge optical emission spectroscopy (GDOES). These techniques have permitted the structural and chemical characterisation of the silver doped hydroxyapatite thin films. The antimicrobial effect of the Ag:HAp thin films on *Escherichia coli* and *Staphylococcus aureus* bacteria was then investigated. This is the first study on the antimicrobial effect of Ag:HAp thin films obtained by sol-gel method. The results of this study have shown that the Ag:HAp thin films with *x*_Ag_ = 0.5 are effective against *E. coli* and *S. aureus* after 24 h.

## 1. Introduction

Nowadays, the preparation and study of inorganic materials at nanometer scale have attracted considerable interest in both fundamental and applied research [1–3]. A wide range of applications for nanoparticles have been found in catalysis, electronics, sensors, high density information storage, luminescence devices, photonics, pharmaceuticals, biotechnology, and medicine [4, 5].

One of the materials most studied for biomedical applications such as implants, coatings, and prostheses is hydroxyapatite (HAp) having the general formula Ca_10_(PO_4_)_6_(OH) due to its outstanding biocompatible properties. Synthetic HAp is currently used as biomaterial for many applications in both dentistry and orthopedics, because it forms a real bond with the surrounding bone tissue when implanted [6, 7].

Human beings are often infected by microorganisms such as bacteria, molds, yeasts, and viruses in the living environment. Research on antibacterial materials containing various natural or inorganic substances [8, 9] has been intensive due to the ability of pathogenic bacteria to resist existing antimicrobial agents [10, 11]. The development of postoperatories infections represents a major health problem and new materials with antibacterial properties are at the top of research facilities worldwide. The bactericidal effect of silver NPs as well as silver nanocomposites or silver nanoparticle-based materials is known and has been intensively studied recently [11–15]. The antibacterial activity of silver-containing materials may help in reducing infections when treating burns [16] as well as in preventing bacterial colonization of prostheses and catheters [17, 18].

A crucial property of HAp is its ability to accept a substitution into its structure of the Ca^2+^ ions by other metal ions such as Ag^+^, Cu^2+^, and Zn^2+^. Thus, by managing to substitute Ca^2+^ ions with Ag^+^ into HAp structure, the newly resulted compound will both be biocompatible and will also have antibacterial properties due to the silver ions from the HAp matrix [19, 20]. Thus, silver doped hydroxyapatite could be successfully used as coating for medical implantable devices in order to reduce the risk of postoperatory infections.

In contrast to extensive studies on silver hydroxyapatite thin films prepared by immersing of the hydroxyapatite thin film in a beaker with AgNO_3_ solution, we presented for the first time silver hydroxyapatite thin films obtained by sol-gel dip coating method. This method is fast, easy to set up, and does not require any vacuum stage. The resultant Ag:HAp (*x*_Ag_ = 0 and *x*_Ag_ = 0.5) films coated on commercially pure Si disks substrates were systematically characterized by scanning electron microscopy (SEM) coupled with X-ray energy dispersive spectroscopy detector (X-EDS), Glow discharge optical emission spectroscopy (GDOES), and Fourier Transform InfraRed spectroscopy (FT-IR). In addition, information on their antimicrobial effect against Gram-negative *Escherichia coli* and Gram-positive *Staphylococcus aureus* was recorded for the first time.

## 2. Experimental Section

### 2.1. Thin Films of Silver Doped Hydroxyapatite

Silver doped hydroxyapaptite (Ag:HAp) coatings on commercially pure Si disks substrate were synthesized by the sol-gel dip coating method. In order to synthesize the Ag:HAp precursors of calcium nitrate (Ca(NO_3_)_2_∗4H_2_O, Aldrich, USA), diammonium hydrogen phosphate ((NH_4_)_2_HPO_4_; Wako Pure Chemical Industries Ltd.) and AgNO_3_ (Alpha Aesare, Germany, 99.99% purity) were used. Controlled amounts of diammonium hydrogen phosphate and silver nitrate were dissolved in ethanol. After adding distilled water, the solution was stirred vigorously for 24 h at 40°C. In a separate container, a stoichiometric amount of calcium nitrate was dissolved in ethanol with vigorous stirring for 24 h at 40°C. The Ca-containing solution was added slowly to the P-containing solution and then aged at room temperature for 72 h and further at 40°C for 24 h. The composition ratios in the Ag:HAp (*x*_Ag_ = 0 and *x*_Ag_ = 0.5) sol were adjusted to have [Ca + Ag]/P as 1.67 [21, 22]. Commercially pure Si disks were ultrasonically cleaned and rinsed in acetone and distilled water prior to being dipped in the Ag:HAp (*x*_Ag_ = 0 and *x*_Ag_ = 0.5) sol. All specimens were dipped into the sol-gel and then they were dried for 2 h at 100°C. The dried coatings were heated in air atmosphere for 3 h at 600°C.

### 2.2. Characterization Methods

The morphology of the material was studied using a Quanta Inspect F scanning electron microscope (SEM). The elemental local analysis of the coatings was performed using an energy dispersive spectroscope (EDS) detector from X-EDS. Operating conditions were an accelerating voltage between 2 and 25 keV (depending on the ratio signal/noise) with samples tilted at 25° to get the optimal take-off angle (30°) allowing a dead time around 20–30% and a collecting time of 90–120 s. The top surface analysis of the samples was studied by glow discharge optical emission spectroscopy (GDOES) [23] using a GD Profiler 2 from Horiba/Jobin Yvon. The technique is suitable for thin film analysis and permits to determine the chemical gradient composition from the surface to the bulk and—if the ablation rate can be estimated—to precise the thickness of the different layers of the nanocomposite materials [24]. The formation and structure of the apatite layer were analyzed using Fourier transform infrared spectroscopy (FT-IR) (Spectrum BX) in the range of 4000–400 cm^−1^ with spectral resolution of 4 cm^−1^.

### 2.3. Antimicrobial Activity

The antimicrobial activity of the silver doped hydroxyapatite thin films was evaluated by the Luria-Bertani agar plate method [25]. A positive control was done using a pure Si disk substrate without any film deposited. The microorganisms used in this study for testing the antimicrobial activities, *Staphylococcus aureus* (*S. aureus 0364*) and *Escherichia coli* (*E. coli ATCC 25922*), were selected as models of Gram-positive and Gram-negative bacteria. *Staphylococcus aureus* (*S. aureus 0364*) and *Escherichia coli* (*E. coli ATCC 25922*) were grown in Luria-Bertani (LB) medium at 37°C using 100 mL flasks filled with 10 mL of the respective medium. Before the microbiological experimentation, all glass wares and samples were sterilized by autoclaving at 120°C for 30 min. To study the antimicrobial activity of Ag:HAp thin films, a suspension containing 10 *μ*L of microbial cells (ca. 10^9^ CFU (colony forming units)/mL) suspended in 1 mL broth solution was made as described by Ferrer et al. [26]. Samples were removed after 0, 6, 12, and 24 h immersed in 20 cm^3^ of sterile saline, and vortexed for 60 s to resuspend the bacteria. A viability count was performed by dilution and plating on LB in triplicate and incubation at 37°C for 24 h. The viable cells were counted by quantization of colony-forming units (CFUs).

## 3. Results and Discussions

**Figure 1:**
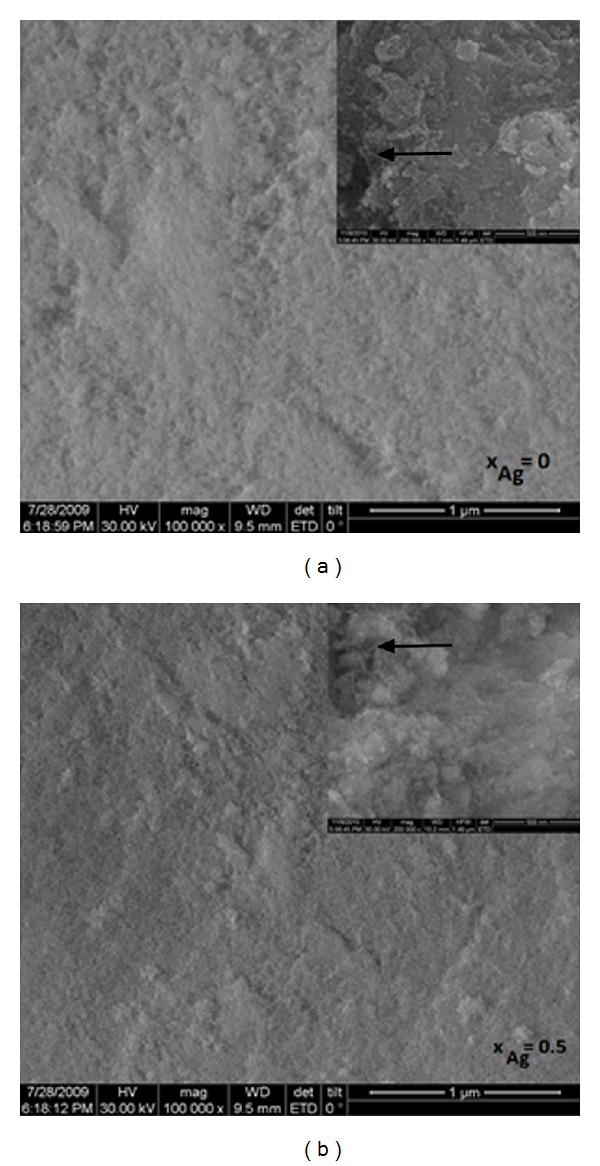
SEM micrographies of the Ag:HAp thin films with *x*_Ag_ = 0 and *x*_Ag_ = 0.5.

**Figure 2:**
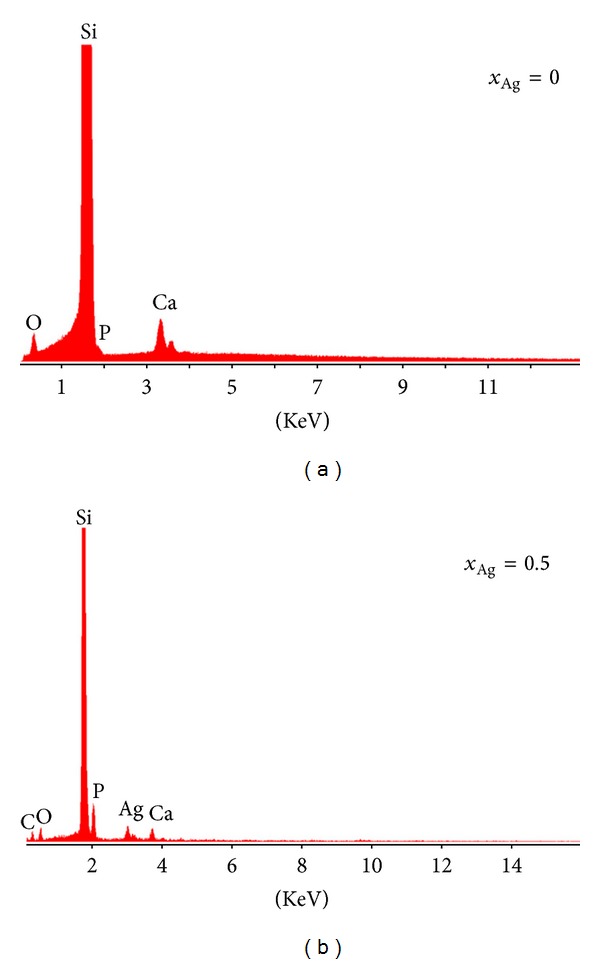
X-EDSX-EDS spectrum of the Ag:HAp thin films with *x*_Ag_ = 0 and *x*_Ag_ = 0.5.

**Figure 3:**
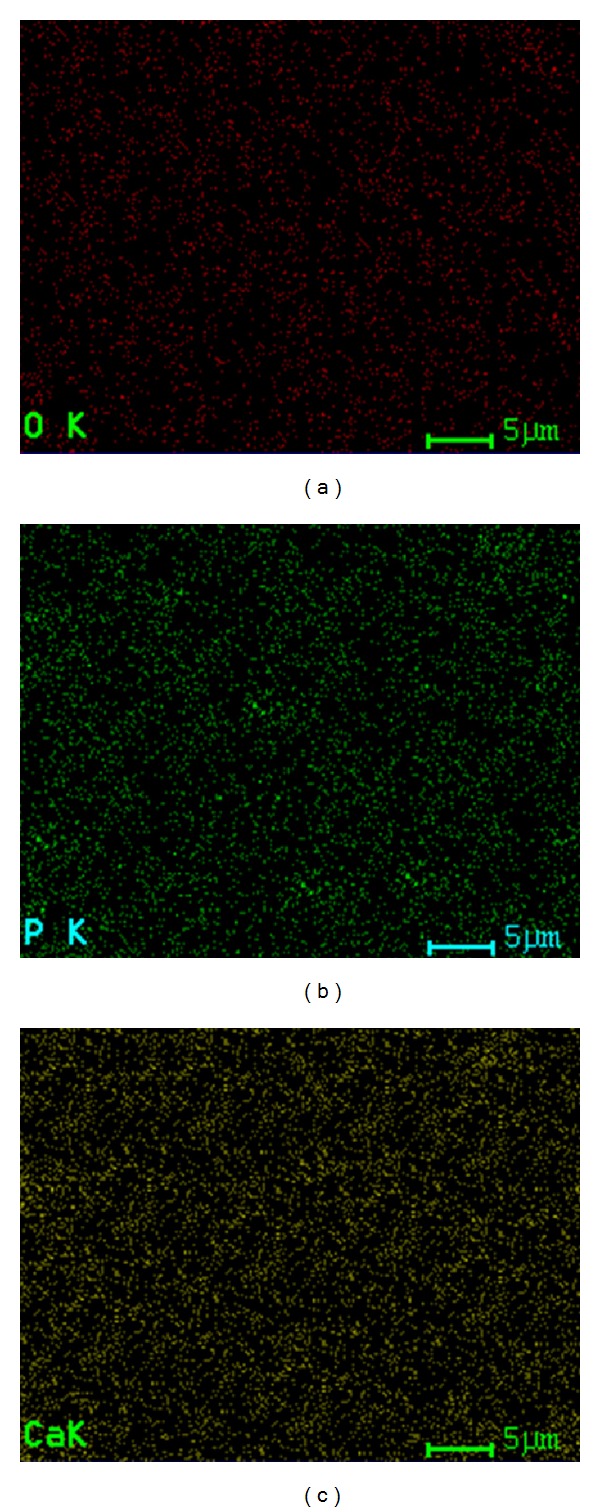
Simultaneous distribution of individual elements of Ag:HAp (*x*_Ag_ = 0) thin films.

**Figure 4:**
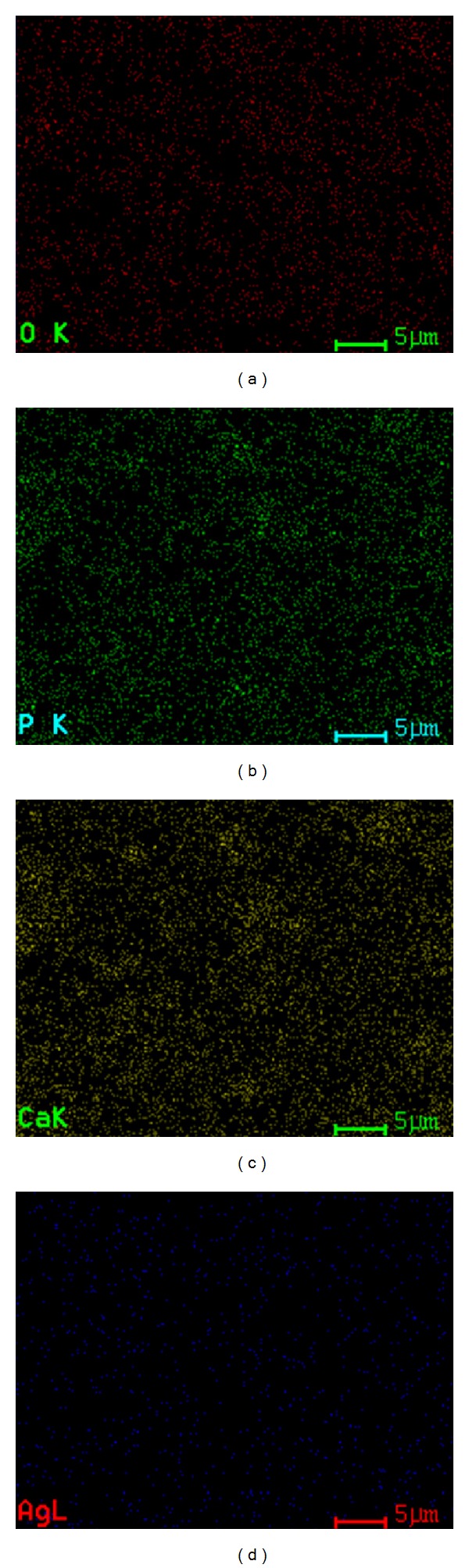
Simultaneous distribution of individual elements of Ag:HAp (*x*_Ag_ = 0.5) thin films.

The sol-gel deposition method offers different advantages such as a very good control of the composition and a good crystallization of HAp films at lower temperature. To investigate the influence of different *x*_Ag_ concentrations on the properties of the Ag:HAp layers, we have comparatively studied Ag:HAp films deposited with *x*_Ag_ = 0 and *x*_Ag_ = 0.5. The elemental composition of the films was examined by both X-EDS and GDOES. The texture of the Ag:HAp films deposited with *x*_Ag_ = 0 and *x*_Ag_ = 0.5 after the heat treatments at 600°C (Figure 1) has been determined by SEM. The surface of Ag:HAp (*x*_Ag_ = 0 and *x*_Ag_ = 0.5) thin films after heat treatment at 600°C is macroscopically homogeneous with a uniform morphology. On the small micrographies embedded in Figure 1, small open pores can be observed (black arrows). Thus, silver hydroxyapatite thin films obtained from composite targets containing Ca_10−*x*_Ag_*x*_ (PO_4_)_6_(OH)_2_ with *x*_Ag_ = 0 and *x*_Ag_ = 0.5 after the heat treatments at 600°C are found to be porous. Weng and Baptista in previous studies [27] have shown that a porous morphology can permit and facilitate the circulation of physiological fluid, when the composite is used for biomedical applications. The results suggest that the doping with Ag^+^ has not drastically influenced the texture of the HAp. The X-EDS (Figure 2) X-EDSX-EDS shows characteristic peaks corresponding to the different elements in the film such as calcium (Ca), phosphor (P), oxygen (O), and silver (Ag) in the Ag doped sample. The Si peak corresponds to the substrate on which the films were deposited. The [Ca + Ag]/P ratio for the stoichiometric Ag:HAp was found to be similar to the previously reported value of 1.67 [22]. The distribution of these elements was homogeneous in the two samples Ag:HAp (*x*_Ag_ = 0 and *x*_Ag_ = 0.5) examined by X-ray elemental mapping analysis (Figures 3 and 4). The X-EDS spectrum mapping of the distribution of individual elements in Ag:HAp thin films (*x*_Ag_ = 0.5) confirmed the presence of silver randomly distributed into the sample. From these data, it is possible to conclude that nondoped and Ag doped thin film materials are laterally homogeneous from both textural and chemical point of view.

Thin films based on hydroxyapatite have been used in various medical fields such as dentistry and orthopedics and act as protective coatings against various influences (bacteria, fungus, etc.). The properties of the films strongly depend on their characteristics, such as the chemical depth profile composition and structure. Dubent et al. [28], Pisonero et al. [29], and Galindo et al. [30] have shown that the optimization of chemical parameters requires adequate analytical techniques with good sensitivity, good reproducibility, and high depth resolution. Many previous studies have demonstrated the capability of GDOES for achieving these requirements.

**Figure 5:**
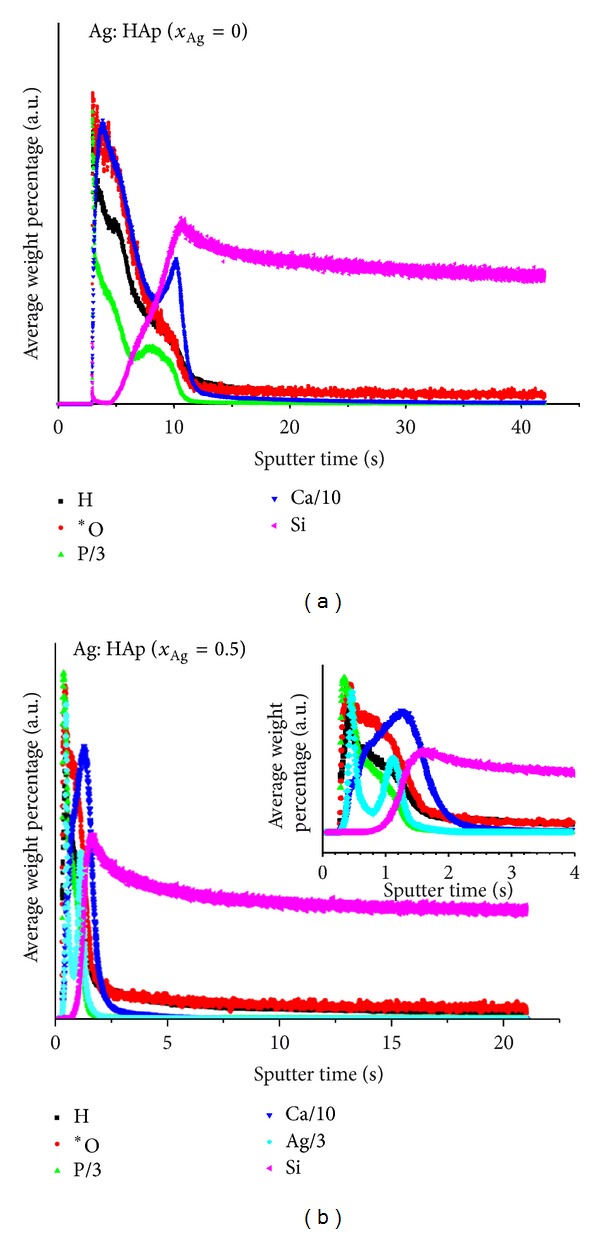
Typical GDOES composition depth profile of silver hydroxyapatite thin films obtained from composite targets containing Ca_10−*x*_Ag_*x*_(PO_4_)_6_(OH)_2_ with *x*_Ag_ = 0 and *x*_Ag_ = 0.5 after the heat treatments at 600°C.

Different GDOES spectra were thus obtained, respectively, performed on various thin films of silver doped hydroxyapatite nanoparticles obtained from composite targets containing Ca_10−*x*_Ag_*x*_(PO_4_)_6_(OH)_2_ with *x*_Ag_ = 0 and *x*_Ag_ = 0.5 and deposited on a pure Si substrate.  Figure 5 shows the GDOES depth profiles after the heat treatments at 600°C. All elements were measured; only the ones presented show a significant signal.

The result reveals the presence of a material composed mainly of calcium, phosphate, oxygen, hydrogen, and silver (*x*_Ag_ = 0.5). The observation of the chemical composition of the coatings with GDOES measurements gives information on the distribution of the elements throughout the film.

The integration of the signals over the entire profile corresponds to the average composition of the target. The GDOES can therefore be used to analyze and optimize the deposition process for functional thin films.

**Figure 6:**
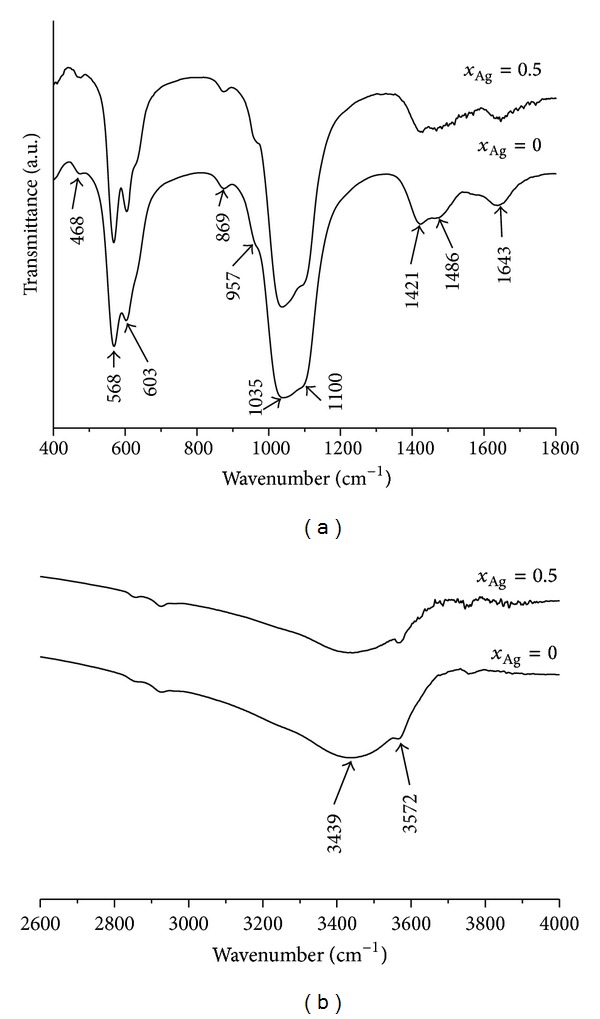
FT-IR spectra of silver hydroxyapatite thin films obtained from composite targets containing Ca_10−*x*_Ag_*x*_(PO_4_)_6_(OH)_2_ with *x*_Ag_ = 0 and *x*_Ag_ = 0.5 after the heat treatments at 600°C.

The structure of the films was investigated by infrared spectroscopy. The FT-IR spectra of the films obtained from composite targets containing Ca_10−*x*_Ag_*x*_(PO4)_6_(OH)_2_ with *x*_Ag_ = 0 and *x*_Ag_ = 0.5 after the heat treatments at 600°C are shown in Figure 6. In the crystalline film, the bands characteristic of PO_4_^3−^ tetrahedral apatite's structure are clearly observed. The peaks in the regions 450–650 cm^−1^ and 950–1100 cm^−1^ are characteristic of a well-crystallized apatite phase. The peaks at 468 and 957 cm^−1^ are assigned to *ν*_2_ stretching and *ν*_1_ bending modes of PO_4_^3−^ and the peaks at 1035 and 1100 cm^−1^ are assigned to *ν*_3_ stretching of PO_4_^3−^ On the other hand the, two peaks at 603 and 568 cm^−1^ are assigned to the *ν*_4_ mode of PO_4_^3−^. Termine and posner [31, 32] and LeGeros [33] in their previous studies have shown that the large separation of the peaks at 568 and 603 cm^−1^ is an indicator of a highly crystallized apatitic phase.

Holmes and Beebe [34] and Layrolle et al. [35] showed that the absorption bands at 1421 and 1486 cm^−1^ come from *ν*_3_ modes of the carbonate and are characteristic of a carbonate in an amorphous phase. The P–OH stretching mode in HPO_4_^2−^ is usually observed at around 870 cm^−1^. Moreover, they showed that the band of 1056 cm^−1^ (assigned to the *ν*_3_ P–O stretching) indicates that the film contains an amorphous phase [34, 35]. The absorption peak in the region of 1600–1700 cm^−1^ ascribed to O–H bending mode is evidence of the incorporated water molecules [35, 36]. The broad bands in the region 3200–3600 cm^−1^ correspond to H–O–H bands of water lattice [37, 38].

The phase detected with FT-IR spectroscopy in silver hydroxyapatite thin films obtained from composite targets containing Ca_10−*x*_Ag_*x*_(PO_4_)_6_(OH)_2_ with *x*_Ag_ = 0 and *x*_Ag_ = 0.5 after the heat treatments at 600°C was hydroxyapatite. Our FT-IR spectra were in good agreement with the data in the literature presented by Stoica et al. and R.Z. LeGeros and J.P. LeGeros [39, 40].

IR spectroscopic analyses have proved that the phase composition of silver hydroxyapatite thin films obtained from composite targets containing Ca_10−*x*_Ag_*x*_(PO_4_)_6_(OH)_2_ with *x*_Ag_ = 0 and *x*_Ag_ = 0.5 after the heat treatments at 600°C is approximately the same. The bands corresponding to the stretching CO_3_^2−^ vibration are presented in the both samples. The intensity of the peak decreases for silver hydroxyapatite thin films with *x*_Ag_ = 0.5. In the previous studies, Ellies et al. [41] showed that the carbonate content of the heated films is also influenced by a number of factors including the starting carbonate content, temperature, and the duration of the heat treatment.

On the other hand, GDOES studies have confirmed the purity of the prepared silver hydroxyapatite thin films obtained from composite targets containing Ca_10−*x*_Ag_*x*_(PO_4_)_6_(OH)_2_ with *x*_Ag_ = 0 and *x*_Ag_ = 0.5. All minor elements measured with the instrument did not show any peak in the profile indicating that their concentration level is low (<0.01% typically). As shown in previous studies realised by Koltsov et al. [42], for nearly all coating technologies, it is important to have clean surfaces prior to the deposition and GDOES offers a quick way to assess this cleanliness.

**Figure 7:**
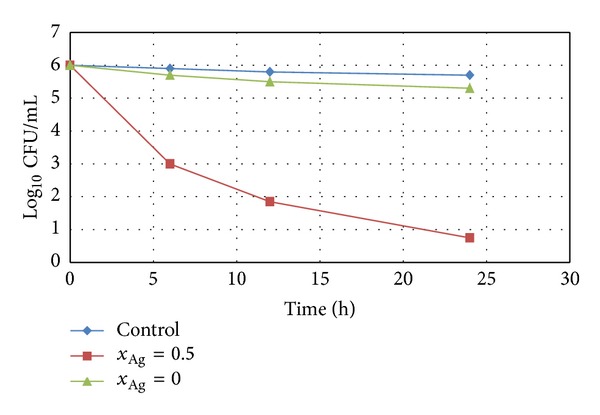
Logarithmic reduction of *E. coli ATCC 25922* population as a function versus time for Ag:HAp thin films with *x*_Ag_ = 0, *x*_Ag_ = 0.5, and control.

**Figure 8:**
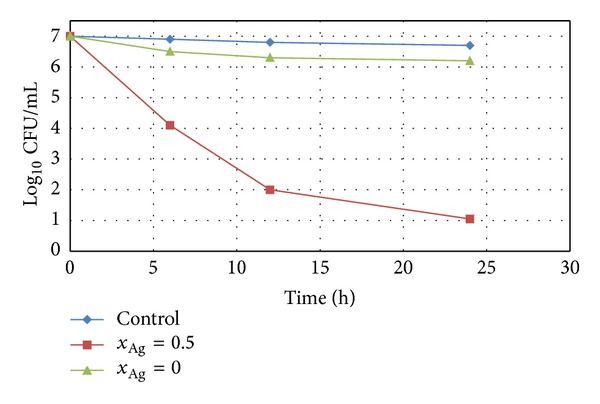
Logarithmic reduction of *S. aureus 0364 *population as a function versus time for Ag:HAp thin films with *x*_Ag_ = 0, *x*_Ag_ = 0.5, and control.

The obtained homogeneous and porous silver doped hydroxyapatite thin films can open new perspectives for the development of antibacterial surfaces to be used in medical process, particularly in the nosocomial (hospital) environment, such as surgery site. For many years, silver has been known as a disinfectant with a broad spectrum of antibacterial activity and low toxicity. On the other hand, the hydroxyapatite has been widely used for biomedical applications due to its good biocompatibility. Previous studies [43] have shown that the cation exchange rate of HAp with silver ions is very high. In order to explore the potential of antimicrobial inorganic materials as protective surfaces against infectious agents, we developed a study to investigate the antimicrobial effect of Ag:HAp thin films prepared by sol-gel method. The antimicrobial effects of the Ag:HAp (*x*_Ag_ = 0 and *x*_Ag_ = 0.5) nanocomposite thin films were investigated against Gram-negative *Escherichia coli* and Gram-positive *Staphylococcus aureus* bacteria, as shown in Figures 7 and 8.

The Ag:HAp thin films with *x*_Ag_ = 0 showed an approximately 1 log reduction in the viability of *E. coli* and *S. aureus* within 24 h. For the Ag:HAp thin films with *x*_Ag_ = 0.5, a 4 log reduction in the viability of *E. coli* within 12 h and a 5 log reduction within 24 h can be noticed (Figure 7). The antimicrobial activity of Ag:HAp thin films with *x*_Ag_ = 0.5 slightly increases in the presence of *S. aureus*. In Figure 8 is presented a 5 log reduction in the viability of *S. aureus* within 12 h and a 6 log reduction within 24 h for the Ag:HAp thin films with *x*_Ag_ = 0.5. A 3 log reduction in viability is observed for both *E. coli* and *S. aureus* after 6 h for the Ag:HAp thin films with *x*_Ag_ = 0.5. The activity of the Ag:HAp thin films with *x*_Ag_ = 0.5 is much better than that encountered in the films without silver even after 6 h. However, the results presented in this study show that the Ag:HAp (*x*_Ag_ = 0.5) thin films are capable of giving a good annihilation activity within a short time, even after 6 h. The changes in the activity of the Ag:HAp thin films with *x*_Ag_ = 0.5 are related to the presence of Ag to the surface.

Unlike HAp thin films, Ag:HAp thin films showed antimicrobial activity against *Escherichia coli* and *Staphylococcus aureus* bacteria. This behavior could be explained by electrostatic forces between the material surface and the bacterial cell membranes [44]. This happens due to the generation of large quantities of hydrophilic functional groups on the surface of Ag:HAp thin films. Similar results were obtained by Murakami et al. [44] and Mo et al. [45] on hydroxyapatite coatings on titanium and silver-hydroxyapatite/titania nanocomposite thin film on titanium. With regard to the antibacterial effect of silver hydroxyapatite/titania thin film, Mo et al. [45] showed that more than 99% of *S. aureus* and *E. coli* were killed after 24 h incubation. In their earlier studies, they have also showed that Ag^+^ inhibits the DNA synthesis, directly binding on the bacterial DNA. Moreover, Ag^+^ also adsorbs the protein on the surface of the bacterial membrane influencing membrane synthesis with S–Ag bonds. On the other hand, in agreement with previous studies conducted by Chua et al. [46], it can be assumed that the antimicrobial activity on Ag:HAp was caused by the surface negative charge associated with the OH hydrophilic functional groups, thereby creating a repulsion force between the surface and the bacterial cells [45, 46].

## 4. Conclusions

In this paper, a simple and low cost methodology to obtain Ag:HAp thin films is described and the characterization of the materials synthetized is presented. The silver hydroxyapatite thin films obtained by sol-gel technique from composite targets containing Ca_10−*x*_Ag_*x*_(PO_4_)_6_(OH)_2_ with *x*_Ag_ = 0 and *x*_Ag_ = 0.5 presented a granular surface morphology. GDOES measurements show that a substantial Ag content has been deposited in the films. The X-EDS and GDOES spectra revealed the presence of a material composed mainly of phosphate, calcium, oxygen, hydrogen, and silver. IR spectroscopy proved that hydroxyapatite was deposited. IR spectroscopic analyses showed that the phase composition of the film derived from precursor solutions with *x*_Ag_ = 0 and *x*_Ag_ = 0.5 is approximately the same. The antimicrobial efficiency of Ag:HAp thin films against *Escherichia coli* and *Staphylococcus aureus* bacteria was demonstrated. Ag:HAp thin films could lead to a decrease of infections especially in the case of bone and dental implants by surface modification of implantable medical devices.

## Conflict of Interests

The authors declare that there is conflict of interest.
